# Correction to: Strategic crossing of biomass and harvest index—source and sink—achieves genetic gains in wheat

**DOI:** 10.1007/s10681-017-2086-y

**Published:** 2017-12-14

**Authors:** Matthew P. Reynolds, Alistair J. D. Pask, William J. E. Hoppitt, Kai Sonder, Sivakumar Sukumaran, Gemma Molero, Carolina Saint Pierre, Thomas Payne, Ravi P. Singh, Hans J. Braun, Fernanda G. Gonzalez, Ignacio I. Terrile, Naresh C. D. Barma, Abdul Hakim, Zhonghu He, Zheru Fan, Dario Novoselovic, Maher Maghraby, Khaled I. M. Gad, ElHusseiny G. Galal, Adel Hagras, Mohamed M. Mohamed, Abdul Fatah A. Morad, Uttam Kumar, Gyanendra P. Singh, Rudra Naik, Ishwar K. Kalappanavar, Suma Biradar, Sakuru V. Sai Prasad, Ravish Chatrath, Indu Sharma, Kishor Panchabhai, Virinder S. Sohu, Gurvinder S. Mavi, Vinod K. Mishra, Arun Balasubramaniam, Mohammad R. Jalal-Kamali, Manoochehr Khodarahmi, Manoochehr Dastfal, Seyed M. Tabib-Ghaffari, Jabbar Jafarby, Ahmad R. Nikzad, Hossein Akbari Moghaddam, Hassan Ghojogh, Asghar Mehraban, Ernesto Solís-Moya, Miguel A. Camacho-Casas, Pedro Figueroa-López, Javier Ireta-Moreno, Jorge I. Alvarado-Padilla, Alberto Borbón-Gracia, Araceli Torres, Yei Nayeli Quiche, Shesh R. Upadhyay, Deepak Pandey, Muhammad Imtiaz, Monsif U. Rehman, Manzoor Hussain, Makhdoom Hussain, Riaz Ud-Din, Maqsood Qamar, Muhammad Sohail, Muhammad Y. Mujahid, Gulzar Ahmad, Abdul J. Khan, Mahboob A. Sial, Pompiliu Mustatea, Eben von Well, Moses Ncala, Stephan de Groot, Abdelraheem H. A. Hussein, Izzat S. A. Tahir, Amani A. M. Idris, Hala M. M. Elamein, Yann Manes, Arun K. Joshi

**Affiliations:** 1 International Maize and Wheat Improvement Center (CIMMYT), Apdo, 6-641, 06600 Mexico, DF Mexico; 2 Leeds University, Leeds, UK; 3 Instituto Nacional de Tecnología Agropecuaria, Pergamino, Argentina; 4 Bangladesh Agricultural Research Institute, Gazipur, Bangladesh; 5 CIMMYT, Beijing, China; 6 Xinjiang Academy of Agricultural Science, Wulumuqi, China; 7 Agricultural Institute Osijek, Osijek, Croatia; 8 CIMMYT, Sohag, Egypt; 9 Field Crops Research Institute, Cairo, Egypt; 10 CIMMYT BISA, Punjab, India; 11 Indian Agricultural Research Institute, New Delhi, India; 12 University of Agricultural Sciences, Dharwad, India; 13 Indian Agricultural Research Institute, Indore, India; 14 Indian Institute of Wheat and Barley Research, Karnal, India; 15 Syngenta India Ltd., Karnal, India; 16 Punjab Agricultural University, Ludhiana, India; 17 Banaras Hindu University, Varanasi, India; 18 CIMMYT, Tehran, Iran; 19 Seed and Plant Improvement Institute, Karaj, Iran; 20 Dryland Agricultural Research Institute, Maragheh, Iran; 21 Instituto Nacional de Investigaciones Forestales, Agrícolas y Pecuarias, Mexico, Mexico; 22 CIMMYT CENEB, Obregon, Mexico; 23 Nepal Agriculture Research Council, Bhairahawa, Nepal; 24 CIMMYT, Islamabad, Pakistan; 25 Regional Agricultural Research Institute, Bahawalpur, Pakistan; 26 Wheat Research Institute, Ayub Agricultural Research Institute, Faisalabad, Pakistan; 27 Crop Sciences Research Institute, National Agricultural Research Council, Islamabad, Pakistan; 28 Cereal Crop Research Institute, Nowshera-Pirsabak, Pakistan; 29 Nuclear Institute for Food and Agriculture, Tarnab-Peshawar, Pakistan; 30 Nuclear Institute of Agriculture, Tando-Jam, Pakistan; 31 National Agricultural Research and Development Institute, Fundulea, Romania; 32 Small Grain Institute, Bethlehem, South Africa; 33 Sensako Pty Ltd, Bethlehem, South Africa; 34 Agricultural Research Corporation, Wad Medani, Sudan; 35 Syngenta, Paris, France; 36 CIMMYT, Kathmandu, Nepal

## Correction to: Euphytica (2017) 213:257 10.1007/s10681-017-2040-z

The original article was corrected. Author Muhammad Kundi should instead read: Muhammad Sohail.

